# Vitamin E Supplementation in Chemical Colorectal Carcinogenesis: A Two-Edged Knife

**DOI:** 10.3390/nu6083214

**Published:** 2014-08-13

**Authors:** Celia Cohen, João Felipe Rito Cardoso, Sergio Britto Garcia, Helio Vannucchi

**Affiliations:** 1 Department of Internal Medicine, Ribeirão Preto Medical School, University of São Paulo, Ribeirão Preto14049-900, Brazil; E-Mail: hvannucc@fmrp.usp.br; 2 Department of Pathology and Legal Medicine, Ribeirão Preto Medical School, University of São Paulo, Ribeirão Preto 14049-900, Brazil; E-Mails: jfcardoso@usp.br (J.F.R.C.); sbgarcia@fmrp.usp.br (S.B.G.)

**Keywords:** vitamin E, colorectal carcinogenesis, aberrant crypt foci, oxidative stress, PCNA, cyclooxygenase-2, experimental

## Abstract

This work investigated the effects of Vitamin E (VE) on aberrant crypt foci (ACF) incidence, oxidative stress parameters (serum and hepatic VE concentration, and homocysteine, glutathione (GSH), and malondialdehyde (MDA) levels), and expression of both cyclooxygenase-2 (COX2) and proliferating cellular nuclear antigen (PCNA) in experimental colorectal carcinogenesis. Male Wistar rats received subcutaneous injections of 1,2-dimethylhydrazine (DMH) twice a week, for two weeks (40 mg/kg), except for the Control group. Animals were separated into groups that received different amounts of VE in the diet: 0 IU (0×), 75 IU (recommended daily intake, RDI), 225 IU (3× RDI), or 1500 IU (20× RDI), during (dDMH) or after (aDMH) administration of carcinogen. The 0×dDMH and 3×dDMH groups showed decreased serum VE levels. Hepatic VE concentration was higher in 3×aDMH as compared with the other groups. All the groups, except the Control and the 0×aDMH groups, had reduced GSH levels. The 0×dDMH, 0×aDMH, and 20×aDMH groups exhibited increased MDA levels. The aDMH groups had higher ACF incidence and PCNA expression. The 0×aDMH group presented higher ACF rate, followed by 20×aDMH. Moreover, the 3×aDMH group displayed reduced ACF incidence and COX2 expression. Multivariate analysis revealed that GSH modulated homocysteine levels and COX2. These results suggested that 1500 IU of VE is hazardous, whereas 225 IU of VE has beneficial effects on chemical colorectal carcinogenesis.

## 1. Introduction

Colorectal cancer (CRC) is the third most common cancer worldwide. It is estimated that over 1.2 million new CRC cases emerged and 608,700 deaths occurred due to this condition in 2008. Australia, New Zealand, Europe, and North America have the highest incidence rates [1].

Diet has been the most studied etiological factor in colorectal carcinogenesis and may account for approximately 50% of the attributable risk in countries with high susceptibility, such as the USA, England, and Australia [2].

Vitamins and minerals have been suggested to influence the risk of developing CRC. Approximately 12% of colon cancer cases have been attributed to the Western-style diet [3]. On the other hand, Mediterranean diets, which are rich in Vitamin E (VE), have been associated with lower incidence of colon cancer [4], and the plasma level of VE has been correlated with reduced risk of having colon cancer [5].

CRC development is a multistage process that involves a number of pathological alterations, from discrete microscopic lesions in the mucosa, such as aberrant crypt foci (ACF), to malignant tumors [6]. ACF are focal colonic mucosa lesions consisting of at least one increased crypt. They are specifically induced by carcinogens and can be considered early morphological markers of colon carcinogenesis [7]. In this context, dimethylhydrazine (DMH) constitutes a complete carcinogen with high specificity for the colon—it initiates carcinogenesis and triggers the promoting steps [8].

Increased cell proliferation is one of the mechanisms that contribute to malignant transformation, so it may serve as a pre-malignant lesion marker [9]. Rapid cell proliferation is a key factor in cancer development, because it is not possible to repair DNA damage fully before cell division takes place [10].

Cyclooxygenases (COX) are key enzymes in prostaglandin synthesis. Overproduction of the isoform type 2, COX2, occurs in multiple stages of colon carcinogenesis [11]. COX2 expression in the tumor tissue acts as a prognostic factor—it is significantly related to histological type, depth of invasion, pathological stage, liver metastasis, lymphatic and venous invasion, TNM stage, and tumor recurrence [12,13].

Reactive oxygen species production occurs under basal conditions, which requires continuous deactivation of these compounds. Increased prooxidant synthesis causes oxidative stress and induces damage in biological molecules. Indeed, these processes have been implicated in a variety of degenerative pathologies, including cancer [14].

VE is a potent inhibitor of human cancer COX2 activity, cell cycle progression, and proliferation [15]; it may slow neoplastic transformation processes in humans [16]. Vitamin supplementation is especially common among the elderly, whereas CRC is usually diagnosed in people over 40 years. Recently, a study showed that dl-Alpha-Tocopheryl Acetate (ATA) supplementation (100, 200, or 300 mg daily) reduces oxidative stress in healthy adults [17]. Hence, the present study aimed to evaluate how different dietary VE (ATA) contents, administered during or after exposure to carcinogen, affects chemically induced colorectal carcinogenesis in rats. Evaluation relied on putative CRC biomarkers such as ACF incidence, oxidative stress parameters, and colonic COX-2 and PCNA expression.

## 2. Experimental Section

This study was approved by the Ethics Committee for Animal Experimentation of Ribeirão Preto Medical School, University of São Paulo, in May 2007, under protocol number 051/2007.

The 80 male Wistar rats used in this work weighed approximately 300 g. The Central Animal Facility of the Ribeirão Preto Medical School, University of São Paulo supplied the animals. The rats were housed in individual stainless steel cages, at 25 °C, in a 12 h light/dark cycle, with ad libitum water intake. The chow was replaced three times a week, and consumption was measured throughout the experiment.

**Figure 1:**
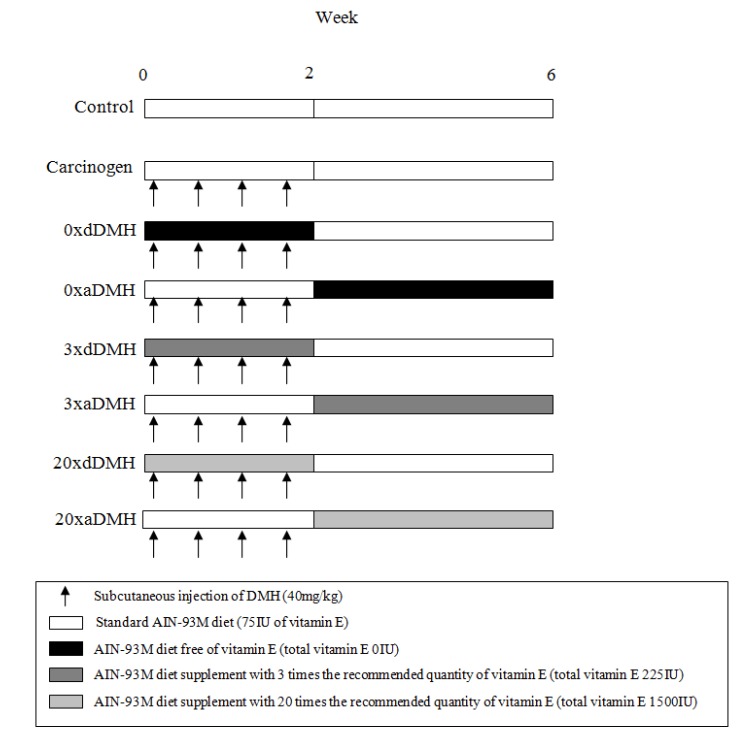
Experimental Design: During the first two weeks, all the groups (except for Control group) received four successive subcutaneous DMH injections. From the beginning until the third week, the 0×dDMH, 3×dDMH, and 20×dDMH groups received diets with different doses of VE (0, 225 IU, and 1500 IU, respectively). Thereafter, these groups received diet with 75 IU of VE up to the end of the experiment. The 0×aDMH, 3×aDMH, and 20×aDMH groups received diets with 75 IU of VE for the first two weeks. Then, from the third week up to the end of the experiment, these groups received diets with different doses of VE (0, 225 IU, and 1500 IU, respectively). The Control and Carcinogen groups received diet with 75 IU of Vitamin E during all the experimental period. All the groups were euthanized at the end of the sixth week. DMH: 1,2-dimethylhydrazine.

After acclimation for one week, the rats were randomly separated into eight groups of 10 rats each. All the groups received four doses of DMH. The exception was the Control group, which received saline injections (see Figure 1).

Animals were euthanized by decapitation 30 days after the fourth DMH injection. Blood was collected in Vacutainer^®^ (BD Company, East Rutherford, NJ, USA) tubes and centrifuged at 2500 rpm for 10 min; serum samples were stored at −70 °C for later analysis. The large bowel was longitudinally excised in the mesenteric border throughout its full extension. The distal colon and rectum were fixed in 10% buffered formalin and embedded in paraffin using methods appropriate for the analysis. For histological (H & E stain) examination of the ACF, samples were embedded in face and sliced (5-μm sections), providing a view of the transversally cut of colonic crypts [18]. For immunohistochemistry, samples were embedded in ring shape maintaining the original transverse orientation of the organ.

### 2.1. Protocol for Experimental Tumor Induction

Symmetrical dimethylhydrazine dihydrochloride (Sigma Co., St Louis, MO, USA) was dissolved in 1 mM EDTA (pH 7.0) immediately before use at a dose of 40 mg/kg of body weight. All the exposed groups received subcutaneous DMH injections twice a week, for two weeks.

### 2.2. Diets

The diet was prepared according to AIN-93M recommendations [19] (Table 1). A vitamin mix was added to the diet in the proportion of 1%, as recommended. dl-Alpha-Tocopheryl Acetate (ATA) 50% powder (C_31_H_52_O_3_, MW = 472.75) was included in the vitamin mix (RHOSTER^®^, São Paulo, Brazil) [20]. All the diet ingredients were weighed, added one by one, and manually mixed with the aid of a strainer. This process was repeated until a homogeneous diet was achieved. Thereafter, the diet was stored at −20 °C. The diet was prepared using soy oil. According to Slover (1971), every 100 g of refined soy oil contains 10.1 mg of alpha-tocopherol (which corresponds to approximately 4.87 IU of alpha-tocopherol/kg of diet [21]), 59.3 mg of gamma-tocopherol, and 26.4 mg of delta-tocopherol [22]. Table 2 lists the final concentration of VE in the diets.

**control group**: Standard AIN-93M diet (75 IU of VE) up to the end of the experiment (six weeks) and subcutaneous saline injection (twice a week, for two weeks)*.*

**Carcinogen group**: Standard AIN-93M diet (75 IU of VE) up to the end of the experiment (six weeks) and subcutaneous DMH injection (40 mg/kg/bw twice a week, for two weeks)*.*

**0**×**dDMH group**: AIN-93M diet, VE-free (0 IU of total VE), during DMH injection.

**0**×**aDMH group**: AIN-93M diet, VE-free (0 IU of total VE), after DMH injection.

**3**×**dDMH group**: AIN-93M diet, VE 3× RDI (225 IU of total VE), during DMH injection.

**3**×**aDMH group**: AIN-93M diet, VE 3× RDI (225 IU of total VE), after DMH injection.

**20**×**dDMH group**: AIN-93M diet, VE 20× RDI (1500 IU of total VE), during DMH injection.

**20**×**aDMH group**: AIN-93M diet, VE 20× RDI (1500 IU of total VE), after DMH injection.

**Table 1:** Composition of the rat diet, based on AIN-93M.

Ingredients	Quantity (%)
Cornstarch	62.25
Casein	14
Soy Oil	4
Sucrose	10
Cellulose	5
Vitamin Mixture	1
Mineral Mixture	3.5
Choline Chloride	0.25

**Table 2:** Composition of the vitamin and mineral pre-mix AIN-93 (Rhoster)/kg of the product.

Composition	ATA Free	Standard	3× ATA	20× ATA
Folic Acid	200 mg	200 mg	200 mg	200 mg
Nicotinic acid	3000 mg	3000 mg	3000 mg	3000 mg
Biotin	20 mg	20 mg	20 mg	20 mg
Calcium pantothenate	1600 mg	1600 mg	1600 mg	1600 mg
Pyridoxine.HCl	700 mg	700 mg	700 mg	700 mg
Riboflavin	600 mg	600 mg	600 mg	600 mg
Thiamine.HCl	600 mg	600 mg	600 mg	600 mg
Vitamin A	400,000 IU	400,000 IU	400,000 IU	400,000 IU
Vitamin B12	2500 mcg	2500 mcg	2500 mcg	2500 mcg
Vitamin D3	100,000 IU	100,000 IU	100,000 UI	100,000 IU
Vitamin E (as dl-alpha tocopheryl acetate)	0 IU	7500 IU	22,500 IU	150,000 IU
Vitamin K1	75 mg	75 mg	75 mg	75 mg
Boron	14.26 mg	14.26 mg	14.26 mg	14.26 mg
Calcium	142.94 g	142.94 g	142.94 g	142.94 g
Chloride	44.9 g	44.9 g	44.9 g	44.9 g
Copper	72.41 mg	72.41 mg	72.41 mg	72.41 mg
Chromium	28.65 mg	28.65 mg	28.65 mg	28.65 mg
Sulfur	8.6 g	8.6 g	8.6 g	8.6 g
Iron	1000 mg	1000 mg	1000 mg	1000 mg
Fluor	28.72 mg	28.72 mg	28.72 mg	28.72 mg
Phosphorus	56.9 g	56.9 g	56.9 g	56.9 g
Iodine	5.93 mg	5.93 mg	5.93 mg	5.93 mg
Lithium	2.85 mg	2.85 mg	2.85 mg	2.85 mg
Magnesium	14.48 g	14.48 g	14.48 g	14.48 g
Manganese	300 mg	300 mg	300 mg	300 mg
Molybdenum	4.32 mg	4.32 mg	4.32 mg	4.32 mg
Nickel	14.31 mg	14.31 mg	14.31 mg	14.31 mg
Potassium	102.86 g	102.86 g	102.86 g	102.86 g
Selenium	4.28 mg	4.28 mg	4.28 mg	4.28 mg
Silicon	143.26 mg	143.26 mg	143.26 mg	143.26 mg
Sodium	29.38 mg	29.38 mg	29.38 mg	29.38 mg
Vanadium	2.87 mg	2.87 mg	2.87 mg	2.87 mg
Zinc	860 mg	860 mg	860 mg	860 mg

### 2.3. Analyses

#### 2.3.1. Oxidative Stress Parameters

Hepatic and serum levels of VE were measured by high-performance liquid chromatography [23]. Briefly, 0.2 g of liver or 500 μL of serum were homogenized in 2.0 mL of ethanol and 1.0 mL of hexane, to extract α-tocopherol. After centrifugation, the hexane layer was transferred to a clean vial, dried under nitrogen stream, reconstituted with mobile phase (methanol/dichloromethane/acetonitrile), and injected into the HPLC system equipped with a column C-18 (Shimpack CLC-ODS 4.6 × 25cm) (Shimadzu Corporation, Tokyo, Japan) and a pre-column measuring 4 mm × 1 cm (Shimadzu Corporation, Tokyo, Japan); the flow rate was 2.0 mL/min. Reading was performed at 292 nm; the final concentration was calculated using an internal α-tocopherol standard (Sigma-Aldrich Company, cod. T3251, St Louis, MO, USA). Hepatic glutathione (GSH) and malondialdehyde (MDA) levels were measured by spectrophotometry [24,25]. To analyze hepatic GSH levels, 200 mg of liver was homogenized in 8.0 mL of EDTA (0.02 M) in ice and 1.0 mL of 50% trichloroacetic acid (TCA). After centrifugation, the supernatant was separated and added to 4.0 mL of TRIS (0.4 M pH 8.9) and 0.1 mL of DTNB (0.01 M in methanol). Absorbance was read at 412 nm, 5 min after stirring with DTNB, against a blank with EDTA (0.02 M). The final concentration was obtained using a standard GSH curve in EDTA (0.02 M). For MDA analysis, 100 mg of liver was homogenized in 1 mL of 1.15% KCl. Then, 2 mL of TBA-TCA-HCl solution was added, and the mixture was heated for 15 min in boiling water bath. After that, the sample was centrifuged for 10 min, at 3000 rpm. The supernatant was separated, and the absorbance was read at 535 nm.

Plasma homocysteine concentrations were obtained by immunoassay using Immulite2000 kit^®^ (Siemens Healthcare, Erlangen, Germany). Briefly, 15 μL of plasma and 300 μL of working solution were homogenized in a tube. Each tube was covered and incubated for 30 min at 37 °C in a water bath. Then, the treated sample was transferred to a cuvette and analyzed.

#### 2.3.2. Aberrant Crypt Foci (ACF)

**Figure 2:**
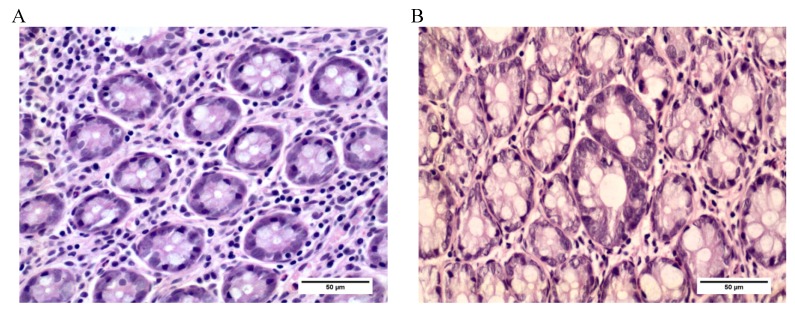
Photomicrography of rat colon tissue sections stained with H & E, ×400. (**A**) Photomicrography of normal colonic crypts. (**B**) Photomicrography of ACF in rats exposed to DMH.

The colonic mucosa was screened to identify, quantify, and analyze the frequency and distribution of ACF, considered early biomarkers of carcinogenesis [6]. After staining with hematoxylin and eosin, microscopic examination of the distal colon mucosa was carried out at 400× magnification; the ACF were identified and quantified, and their frequency per unit of area (cm^2^) was calculated [18] (Figure 2).

#### 2.3.3. Immunohistochemistry

To estimate colonic cyclooxygenase-2 (COX2) expression, 4-μm slices of the paraffin-embedded colon were immunostained with an antibody against COX2, clone 4H12, dilution 1:200 (NCL-COX2/NOVOCASTRA, Leica Biosystems Newcastle, Ltd., Newcastle, UK). One hundred colonic crypts and surrounding connective tissue were observed in each colon. The results for COX2 index (iCOX2) were expressed as positive cell number per colonic crypt [18].

To estimate epithelial cell proliferation, colonic mucosa was immunostained with an antibody against proliferating cell nuclear antigen, clone PC10, dilution 1:100 (NCL-PCNA/NOVOCASTRA, Leica Biosystems Newcastle, Ltd., Newcastle, UK). One hundred colonic crypts nuclei were observed in each colon, and the PCNA index (iPCNA) was estimated as the ration positively marked nucleus/total nucleus ratio per crypt [18].

### 2.4. Statistical Analysis

Data are expressed as mean ± SEM. ANOVA test was used to determine significant differences among the groups, regardless of the Tukey’s Multiple Comparison Test for parametric analysis (weight gain, daily ingestion, and serum and hepatic VE, GSH, MDA, HCY, ACF, and iCOX2 levels) or Kruskall-Wallis Test with Dunn for non-parametric analysis (PCNA-Li). A multivariate analysis was performed to evaluate the effect of: (1) MDA, GSH, hepatic VE, homocysteine, iPCNA, and COX2 on ACF; (2) MDA, GSH, hepatic VE, and COX2 on homocysteine; (3) MDA, GSH, and hepatic VE on COX2; and (4) MDA, hepatic VE, and homocysteine on GSH. The significance level was set at *p* < 0.05 for all the analyses. The software programs SAS^®^ 9 (SAS Institute Inc., Cary, NC, USA) and Graphpad Prism 4.0 (GraphPad Software Inc., La Jolla, CA, USA) were used.

## 3. Results

### 3.1. Daily Ingestion and Weight Gain

**Figure 3:**
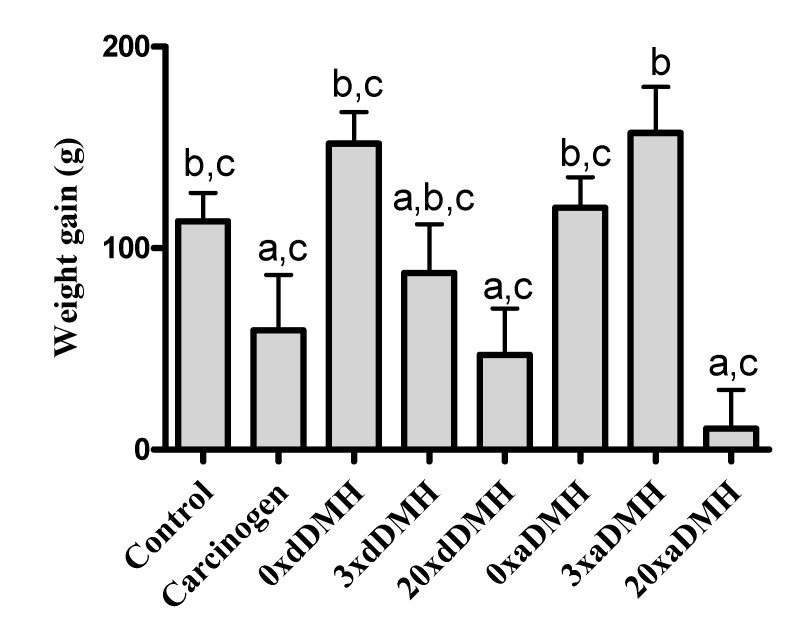
Mean weight gain (g) at the end of the experiment. Bar values with the same superscript letters are not significantly different.

The studied groups did not differ significantly in terms of daily ingestion, but the 20×aDMH group showed reduced ponderal weight gain during the experiment as compared with the Control (*p* < 0.05), 0×dDMH (*p* < 0.001), 0×aDMH (*p* < 0.01), and 3×aDMH (*p* < 0.001) groups. The 20×dDMH group gained less weight as compared with the 0×dDMH (*p* < 0.01) and 3×aDMH (*p* < 0.01) groups. The 3×aDMH group gained more weight as compared with the Carcinogen group (*p* < 0.05) (Figure 3). Necropsy conducted in the presence of a veterinary doctor evidenced reduced adipose tissue around the kidney in the 0×DMH group. The 20×aDMH group did not present any sign of adipose tissue in this area, but it showed signs of severe muscle and adipose tissue loss.

### 3.2. Oxidative Stress Parameters

**Figure 4:**
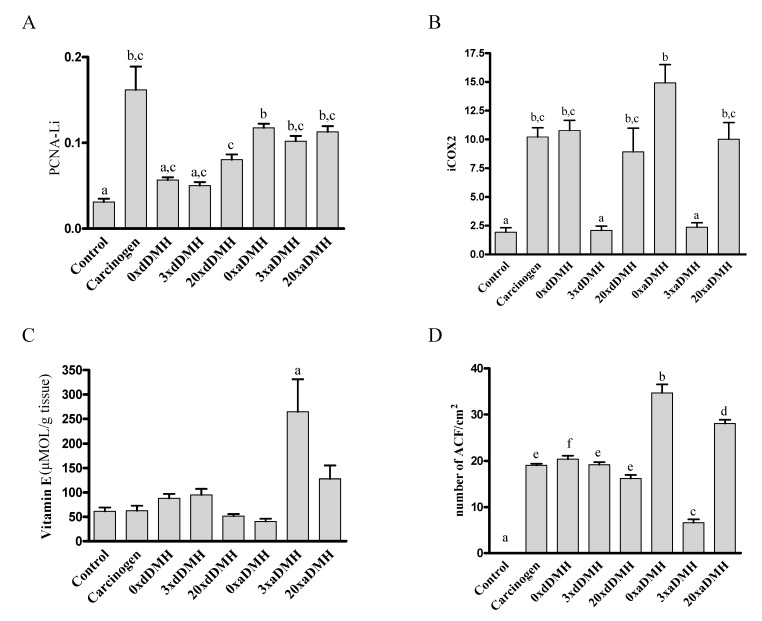
Colonic expression of PCNA and COX-2 obtained by immunohistochemistry, hepatic content of VE measured by HPLC, and ACF count obtained by H & E. Statistical analysis. (**A**) PCNA labeling index (PCNA-Li). (**B**) Cyclooxygenase 2 index (iCOX-2). (**C**) Hepatic content of VE. (**D**) Number of ACF. ^a^ *p* < 0.05 compared to Carcinogen, 0×aDMH, 3×aDMH, 20×aDMH; ^b^ *p* < 0.05 compared to Carcinogen, 0×aDMH, 3×aDMH, 20×aDMH and 20×dDMH; ^c^ *p* < 0.05 compared to Carcinogen, 0×dDMH, 0×aDMH, 20×dDMH and 20×aDMH; ^d^ *p* < 0.05 compared to 0xaDMH; ^e^ *p* < 0.05 compared to other groups; ^f^ *p* < 0.05 compared to 0×dDMH.

Groups receiving VE-free (0×aDMH and 0×dDMH) diet and the 3×dDMH group presented reduced serum VE concentration. The 3×aDMH group had lower serum levels of VE than the Carcinogen group (*p* < 0.05). The 20×dDMH group presented increased VE concentration as compared with the 0×aDMH and 3×dDMH groups (*p* < 0.01) (Table 3). Hepatic VE content was higher in the 3×aDMH group as compared with the other groups (*p* < 0.01) (Figure 4C).

Except for the Control and 0×aDMH group, all the groups exhibited reduced hepatic GSH levels as compared with the Carcinogen group (*p* < 0.05). Groups receiving 20×RDI VE (1500 IU, 20×dDMH, and 20×aDMH) showed decreased GSH levels as compared with the Control group (*p* < 0.05) (Table 3).

The 0×dDMH, 0×aDMH, and 20×aDMH groups presented increased hepatic MDA levels (*p* < 0.05). The 0×dDMH group had lower serum homocysteine concentration than the 20×aDMH group (*p* < 0.05) (Table 3).

### 3.3. Aberrant Crypt Foci

Diet modification after exposure to carcinogen resulted in higher incidence of ACF. The 0×aDMH group had the highest number of ACF (*p* < 0.001), followed by the 20×aDMH group (*p* < 0.001). The 3×aDMH group presented the lowest incidence of ACF (*p* < 0.001) (Figure 4D).

### 3.4. Immunohistochemistry Findings

Administration of the carcinogen and diet modification after exposure to the carcinogen increased iPCNA expression (*p* < 0.05). Compared with the Control group, supplementation in the 20×dDMH group elevated iPCNA (*p* < 0.001) (Figure 4A).

The Control, 3×dDMH, and 3×aDMH groups exhibited reduced iCOX2 expression (*p* < 0.01) (Figure 4B).

### 3.5. Multivariate Analysis

Multivariate analysis showed that increased hepatic GSH content reduced plasma homocysteine levels (estimate −0.08 CI [−0.14; −0.02]). The rise of one unit in GSH levels increased iCOX2 by 0.11 units (estimate 0.11 CI [0.01; 0.21]). Increased hepatic MDA levels slightly raised GSH levels (estimate 0.04 CI [0.01; 0.26]).

**Table 3:** Hepatic and serum levels of biochemical oxidative stress biomarkers in rats submitted to different treatments.

Analysis	Control	Carcinogen	During (dDMH)			After (aDMH)		
			0×	3×	20×	0×	3×	20×
Serum VE (umol/L)	18.37 ± 0.83	19.35 ± 1.75	11.83 ± 0.42 ^a,b,c^	9.50 ± 1.14 ^a,b,c^	16.85 ± 1.47 ^d,e^	7.03 ± 0.46 ^a,b,c^	11.85 ± 1.06 ^b^	19.80 ± 3.22
GSH (umol/gptn)	36.34 ± 1.97	45.95 ± 4.29	25.89 ± 1.99 ^b^	30.90 ± 2.53 ^b^	19.20 ± 1.45 ^a,b^	29.29 ± 3.66	26.37 ± 2.21 ^b^	28.72 ± 3.02 ^b^
MDA (nmol/mgptn)	0.13 ± 0.00	0.13 ± 0.01	0.17 ± 0.01 ^a,e^	0.12 ± 0.01 ^c^	0.15 ± 0.00 ^d^	0.19 ± 0.00 ^a,b,e^	0.15 ± 0.01 ^d^	0.160 ± 0.01 ^a,b^
HCy (umol/L)	6.00 ± 0.32	4.90 ± 0.93	6.65 ± 0.70 ^c^	4.20 ± 0.58	5.05 ± 0.36	5.55 ± 0.70	5.00 ± 0.40	3.90 ± 0.32

VE = vitamin E; GSH = glutathione; MDA = malondialdehyde; HCy = homocysteine. Data are presented as mean ± SEM. ^a^ *p* < 0.05 compared with the Control group; ^b^ *p* < 0.05 compared with the Carcinogen group; ^c^ *p* < 0.05 compared with 20× After; ^d^ *p* < 0.05 compared with 0× After; ^e^ *p* < 0.05 compared with 3× During.

## 4. Discussion

The present study has been the first to investigate how VE (ATA) intake (0 IU, 75 IU, 225 IU, or 1500 IU) during or after exposure to DMH affects chemical colorectal carcinogenesis, as analyzed by indirect (oxidative stress, cell proliferation, and COX2 expression) and direct (incidence of ACF) parameters. Because tissue and serum levels of alpha-tocopherol are higher than the levels of other tocopherols, we decided to use this isoform in the study. Another reason for our choice was the fact that alpha-tocopherol is the isoform that usually occurs in commercial supplements consumed by the general population without regular medical or nutritional monitoring, which could cause serious health impacts.

This study showed that exposure to the carcinogen alone did not affect the serum and hepatic levels of VE. VE (ATA) supplementation at 3× RDI (225 IU) increased hepatic VE storage and reduced the serum values of this vitamin, suggesting the existence of tissue, serum, and peripheral control of VE. These findings agreed with previous studies, suggesting that VE needs protection from other antioxidant systems to prevent its depletion and loss of its regulatory properties. Lack of oxidative mechanisms compensation culminates in VE waste, and VE oxidation products arise [26].

On the other hand, exposure to the carcinogen decreased the hepatic GSH content. GSH is the major regulator of oxidative stress; low GSH level has been associated with cancer pathogenesis [27]. Because of its dual ability to directly scavenge radicals and donate electrons in enzyme-mediated reduction reactions, GSH is the most versatile intracellular antioxidant. Moreover, GSH holds cellular buffer, which is critical for cell proliferation, apoptosis, and maintenance of protein structure [28].

VE (ATA) depletion and 20× RDI supplementation (1500 IU) after exposure to carcinogen augmented hepatic MDA levels. Advanced lipid peroxidation end products, such as MDA, emerged as a consequence of VE insufficiency. These end products may impact cell homeostasis and modify gene expression [29]. A recent study has shown that VE plays a complex role during early stages of lipid peroxidation and may not act as an antioxidant, failing to prevent chain cleavage [30]. A randomized clinical trial has demonstrated that *d*-alpha tocopherol supplementation in healthy adults does not benefit lipid peroxidation [31], which corroborates our findings. Some *in vitro* studies have suggested that high alpha-tocopherol levels have a paradoxical role in LDL autoxidation, causing VE-mediated peroxidation [32,33].

Diet modification after exposure to carcinogen greatly impacts the incidence of ACF. Here, the group receiving VE-free diet after DMH exposure (0×aDMH group) presented the highest number of ACF, followed by the 20×aDMH group. The 3×aDMH group had decreased incidence of ACF. Literature works have reported both inhibition of [34] and increase in [35] chemically induced colonic tumors in VE-supplemented mice. Cook *et al.* (1980) [34] demonstrated that, after exposure to DMH, mice fed with a VE-rich diet (600 mg of DL-alpha tocopheryl acetate/kg of diet) had fewer tumors and colorectal carcinomas than those fed with diet bearing low VE content (10 mg/kg of diet). Sumiyoshi *et al.* (1985) [36] described that VE supplementation (100 mg/kg of diet) delayed the appearance but did not affect the incidence or multiplicity of colon tumors induced by DMH in rats as compared with low VE supplementation (<5 mg/kg of diet).

Most studies on colorectal adenoma have shown that VE exerts a protective effect, suggesting that it acts early in tumorigenesis [37]. The present investigation demonstrated that ingestion of a VE-supplemented diet at 3× the RDI during the early stages of carcinogenesis (dDMH and aDMH) protected against cell proliferation, colonic COX2 expression, and ACF formation, indicating that individuals might need a little higher intake of this vitamin in this situation.

The present work also found that administration of carcinogen and diet modification after exposure to carcinogen resulted in higher iPCNA expression. Tumorigenesis is a multistep process; one of the controversial mechanisms contributing to the malignant transformation is increased cell proliferation, considered a marker of pre-malignant lesions [9]. Rapid cell proliferation is a key factor in cancer development, because it might not be possible to repair DNA damage prior to cell division [10]. Evidence exists that increased epithelial turnover level in the colon is associated with higher risk of colon cancer. Raised colonic cell proliferation occurs in the presence of cancer-promoting factors [38]. An anti-initiation role has been proposed for VE, but little has been the evidence for or against its anti-promotion role. A small controlled clinical study that evaluated how Vitamin A, C, and E supplementation affected patients with colorectal adenomas revealed significantly reduced colonic epithelial cell proliferation in the upper colonic crypt portion (40%) [39]. To date, only a small amount of data is available on how VE impacts colonic cell proliferation, particularly in humans. VE can abate initiation and colonic epithelial cell proliferation, and this is not inconsistent with the fact that supplementation (especially VE) is a relatively recent habit of the population.

The present study showed that the group receiving VE (ATA) supplementation at 3× RDI displayed reduced iCOX2 expression. COX2 overexpression occurs in a variety of malignant diseases and induces cell growth by stimulating proliferation and angiogenesis [40]. In the form of gamma-tocopherol, VE is able to inhibit COX2 activity, cell cycle progression, and human cancer proliferation by downregulating cyclins [41]. A study described that VE (ATA) at 10uM in a culture of Caco2 cells inhibited COX2 activity in a post-transcriptional way [42]. The COX2 results may have influenced weight gain during our experiment, because groups with higher COX2 expression gained less weight. Groups receiving VE 3× (3×dDMH and 3×aDMH) exhibited lower cellular proliferation and COX2 colonic expression, which are important biomarkers of CRC.

Although the 3×dDMH group did not display lower incidence of ACF, our findings suggested that 225 IU of VE might have an anti-initiation and/or anti-promotion role in chemical colorectal carcinogenesis.

The multivariate statistical analysis showed that elevated hepatic GSH levels increased iCOX2 and decreased plasma homocysteine and hepatic MDA levels. These findings indicated that oxidative stress has an important role in chemical colorectal carcinogenesis—it interferes with early carcinogenesis biomarkers, like COX2, which reinforces the need to maintain oxidative balance.

The formation of reactive oxygen species is a normal consequence of a variety of essential biochemical reactions, and excess oxygen radicals can originate in chronic diseases of the gastrointestinal tract. With the progression of the disease, the production of these radicals increases, to culminate in enhanced lipid peroxidation as a result of cell membrane degeneration and DNA damage. Autoxidation can take place in the presence of high homocysteine concentrations, to produce highly detrimental and reactive hydrogen peroxide (H_2_O_2_) [43]. Therefore, high homocysteine concentrations may also have a genotoxic role [44]. In turn, MDA is mutagenic [45], and its reaction with deoxyguanosine produces the main adduct found in DNA from human tissues [46]. As for GSH, it is a versatile reducer that works in various physiological functions, such as radical scavenging by direct reaction, reduction of the enzyme-mediated degradation of hydrogen peroxide and lipid peroxides, maintenance of protein thiol groups, and conjugation and excretion of electrophilic xenobiotics; it also acts as a coenzyme [28]. In the particular case of VE, numerous studies have clarified its prooxidant effect and shown that it is a potential anticancer agent at high doses. These effects are a consequence of alpha-tocopheroxyl radical production, which promotes oxidative stress. High doses of VE may displace other lipid soluble antioxidants (e.g., gamma-tocopherol), to disrupt the natural balance of antioxidant systems and increase vulnerability to oxidative damage. This process involves reactive oxygen species and leads to cancer initiation and promotion. VE may also inhibit human cytosolic glutathione *S*-transferases, which are enzymes that aid detoxification of drugs and endogenous toxins underlying oxidative stress [47].

This study showed that VE (ATA) acts in different pathways of colorectal carcinogenesis: it affects cell growth, inflammation, oxidative stress, and cell death. Gene expression regulation, cellular stress, and protein kinase C (PKC) activity through VE could be the mechanisms involved in this process. Some authors have shown that VE (alpha-tocopherol) can inhibit PKC activity [48,49], modulate gene expression [50], and inhibit cytotoxic response, irrespective of its antioxidant activities [51]. PKC participates in cell growth, death regulation, and stress responsiveness. Moreover, it is a sensitive target for redox modification—it can be activated by oxidative stress and inhibited by antioxidants [52] such as VE and GSH.

## 5. Conclusions

In the current study, we have employed different doses of DL-Alpha-Tocopheryl Acetate (ATA) at different times of exposure to carcinogen to investigate how Vitamin E affects the early stages of colon carcinogenesis. We have shown that oxidative stress participates in the process of cancer initiation and promotion. Diet modification after exposure to carcinogen increased PCNA and COX2 expression. Supplementation at 20× the recommended daily intake (1500IU) was as hazardous as the absence of Vitamin E (ATA), as judged from the increase in the incidence of ACF. Supplementation at three times the recommended daily intake lowered the incidence of ACF, cell proliferation, and COX2 expression, showing that supplementation with 225 IU has a protective role during the early stages of colorectal chemical carcinogenesis in rats.
